# Enterprise-Based Participatory Action Research in the Development of a Basic Occupational Health Service Model in Thailand

**DOI:** 10.3390/ijerph20085538

**Published:** 2023-04-17

**Authors:** Kankamol Passaranon, Naesinee Chaiear, Napak Duangjumphol, Penprapa Siviroj

**Affiliations:** 1 Department of Community, Family, and Occupational Medicine, Faculty of Medicine, Khon Kaen University, Khon Kaen 40002, Thailand; 2 Maharat Nakhon Ratchasima Hospital, Nakhon Ratchasima 30000, Thailand; 3 Department of Community Medicine, Faculty of Medicine, Chiang Mai University, Chiang Mai 50200, Thailand

**Keywords:** occupational health service, in-plant occupational health service, basic occupational health service model, occupational health need, ILO C161 Convention, participatory action research, education and learning experience

## Abstract

Various basic occupational health services (BOHS) are provided, particularly in-plant BOHS; however, it might be necessary to start expanding BOHS. The current study focuses on BOHS model development using participatory action research (PAR) at a large-sized enterprise in northeastern Thailand. The PAR began with a situation analysis using ILO Convention C161, problem and cause analysis, the development of an action plan, observation and action, evaluation, and replanning. The research tools included interviews, focus group discussions (FGDs), and participant observations. The participants included managers, human resource staff, safety officers, and workers. Both inductive and deductive thematic analyses were undertaken. The results showed that (1) education and learning experience led to the workers detecting work-related diseases early by themselves and the implementation of medical surveillance programs; (2) the workers’ occupational health needs led to return-to-work assessments and first aid room system development; (3) the employer’s experience led to appropriate fit-for-work examinations and emergency preparedness; and (4) the feedback from BOHS providers led to a hospital-to-in-plant return-to-work conversion. The study concluded that the enterprise could develop fit-for-work and return-to-work assessments as per the ILO Convention C161 under the policy; however, medical surveillance and the first aid room system need to be developed through counseling at the hospital’s occupational medicine clinic.

## 1. Introduction

The International Labor Organization (ILO, Geneva, Switzerland) estimates that 2.9 million men and women die annually due to illnesses or accidents at work. In addition, 402 million workers worldwide suffer non-fatal occupational injuries [1]. In Thailand, the number of employers increased by approximately 15% between 2017 and 2021 (371,432 vs. 436,817, respectively). The respective number of employees in 2017 and 2021 was 9,777,751 and 11,172,844—an increase of approximately 12%. Remarkably, despite the rise in employers and employees, the number of occupational injuries or illnesses on record at the Social Security Office decreased from 86,278 to 78,245 between 2017 and 2021, and the number of occupational injuries or diseases similarly decreased [2]. Thus, in the face of increasing industrial activities in the last two decades, which introduce new hazards and new health outcomes [3], one way to address the occupational health issues of the working population is to strengthen the provision of occupational health services (OHS) and in particular BOHS [4].

The ILO OHS Convention 161 defines OHS as, “services entrusted with essentially preventive functions and responsible for advising the employer, the workers, and their representatives in the undertaking on the requirements for establishing and maintaining a safe and healthy working environment which will facilitate optimal physical and mental health regarding work and the adaptation of work to the capabilities of workers in the light of their state of physical and mental health.” Therefore, ILO Convention C161 was ratified to protect workers against sickness, disease, and injury from his/her employment [4,5]. Any OHS program is thus established to prevent work-related injuries and illnesses [6]. As such, OHS have beneficial impacts on the workplace with respect to protecting worker health, promoting health, mental well-being, and workability, and the prevention of illness and accidents [7,8].

Notwithstanding good intentions, OHS are unequally distributed and vary from country to country, depending on ILO C161 ratification [9]. OHS coverage is high (75% to 97%) in countries that have ratified ILO C161, such as Croatia, Finland, and Macedonia. In comparison, OHS coverage in countries that have not ratified ILO C161 is low, ranging between 5% and 10% (i.e., China and India) [5,10,11,12]. The estimated global coverage of OHS is 18.8%, so 80% of the world’s working population does not have access to OHS [13]. Thailand’s OHS coverage is inconsistent and systematic assessments are limited. One survey of OHS coverage at provincial public health offices and primary care units revealed that OHS activities ranged between 16 and 100 percent. Still, the results are not generalizable owing to the limited sample [14].

OHS in Thailand is administered by three government ministries: the Ministry of Labor, the Ministry of Public Health, and the Ministry of Industry. At the intermediate level, Thailand has no agencies responsible for supporting occupational health services including (1) providing educational and training programs for occupational health professionals’ qualification, certification, and competencies; (2) occupational health service standards regulated by law; and (3) quality evaluation methods for occupational health services in enterprises. At the local level, there are no specific occupational health service organizations at the organizational level mandated by laws and regulations in Thailand. The Ministry of Public Health developed OHS standards to audit occupational health professionals that provide OHS to enterprises. The Ministry of Labor would not enforce OHS standards, and no OHS recommendations exist for enterprises [15].

Since Thailand has not ratified the ILO C161 Convention [16], Thai laws concerning OHS functions and OHP duties are incomplete. Developing a BOHS model would identify BOHS provision in the workplace and identify the roles of OHP in Thailand. The future of OHS laws remains unpredictable, and an enterprise-concerning policy may be an additional OHS provision in the workplace. PAR was deemed appropriate in this study because it helps the participants to understand the causes of problems that lead to the development of action plans for sustainable problem-solving, on-the-job BOHS provision, and identifying the functions of OHP. Although Thai laws have not mandated using BOHS, applying the PAR model will be essential to fostering internal cooperation. Once the Thai law requires BOHS, the PAR model will quickly proceed along with other Thai laws. Therefore, the research question is, ‘How will an in-plant model of basic occupational health services in workplaces be?’ A company was chosen to develop a BOHS model in Thailand to identify and understand the causes of issues leading to expanding the BOHS model in Thailand. Generally, other enterprises could modify the BOHS model based on occupational health concerns. The current study aimed to use PAR to establish an OHS model in a large-sized enterprise in northeastern Thailand.

## 2. Literature Review

### 2.1. Occupational Health Service Model

The occupational health service models at the enterprise level have been classified depending on places that provide occupational health services, for instance, an in-plant model, an inter-enterprise model, an industry-oriented model, hospital outpatient clinics, private health centers, primary health care units, and a social security model.

The in-plant model was applied in large enterprises to provide occupational health services. Both full ranges of occupational health services and non-occupational health services were provided by multidisciplinary staff such as occupational physicians, occupational nurses, occupational hygienists, ergonomists, toxicologists, occupational physiologists, laboratory and x-ray technicians, physiologists, social workers, health educators, counselors, and industrial psychologists. Smaller enterprises provided occupational health services by one or more full-time occupational health nurses and a part-time occupational physician who prepared standing orders for procedures, medication, and visits as necessary. In addition to this, enterprises connected with external service suppliers to provide in-plant specialized occupational health services (i.e., occupational hygiene, toxicology, and safety engineering). 

Other occupational health service models cannot provide a full range and high quality of services due to a lack of familiarity with the workplace and the limitations of occupational health personnel [6].

This research will apply the in-plant model to developing occupational health services in the workplace because this research aims to develop occupational health services in the workplace as a model. Therefore, the occupational health service model should have the greatest coverage and highest quality of the occupational health service model.

### 2.2. Development of Occupational Health Services at the National Level

The occupational health service infrastructure system is divided into three classes: national, intermediate, and local. Each infrastructure level has different objectives, as shown in Table 1; the national level regulates laws and policies, the intermediate level supports services, and the local level follows the service provisions [17,18].

At the national level, Finland’s authority responsible for regulation and policy is the Ministry of Social Affairs and Health (MoSAH), Malaysia’s is the Ministry of Human Resources (MoHR), and in Vietnam, it is the Ministry of Health (MoH). At the intermediate level, the agencies for support services, for example, training, consultation, certification, research, and development, are in Finland, the Finnish Institute of Occupational Health (FIOH), in Malaysia, the National Institute of Occupational Safety and Health (NIOSH), and in Vietnam, the National Institute of Occupational and Environmental Health (NIOEH). Lastly, at the local level, the occupational health service infrastructure at the organizational level comprises laws or regulations, collective agreements between employers and employees, and organizational personnel. Depending on the company size, service provision agencies in Finland and Malaysia are specific occupational health providers. In contrast, there are no particular organizations in Vietnam. Table 2 compares the organizations responsible for occupational health services in Finland, Malaysia, and Vietnam [4,5,19,20,21,22].

### 2.3. Development of Occupational Health Services in Thailand 

At the national level, Thailand’s occupational health services infrastructure includes the Ministry of Labor, the Ministry of Public Health, and the Ministry of Industry. The Ministry of Labor controls social security to promote compensation for work-related (Workmen Compensation Fund) and non-work-related diseases (social security). The Department of Labor Protection and Welfare promotes workplace safety, regulates companies, and supports academic institutions. The Ministry of Public Health regulates the Department of Disease Control, the Department of Medical Service, and the Office of the Permanent Secretary. The Department of Disease Control provides secondary occupational health services in hospitals. The Department of Medical Service provides tertiary occupational health services (Nopparatrajathanee Hospital). The Office of the Permanent Secretary provides the Provincial Public Health Office and the Primary Care Unit. The Ministry of Industry publishes Regulation of Ministry of Industry No. 4409 (B.E.2555), including the Guideline for Examination Due to Occupational Chemical and Physical Hazards in Workplaces [21].

At the intermediate level, Thailand has no agency responsible for supporting occupational health services (1) to provide educational and training programs for occupational health professionals’ qualification, certification, and competencies; (2) occupational health service standards regulated by law; and (3) quality evaluation methods for occupational health services in enterprises. 

At the local level, there are no specific occupational health bodies services at the organizational level mandated by laws and regulations in Thailand. Instead, the Thailand OHS model comprises a tertiary referral center, in-house OHS, and public occupational health clinic [14,23,24].

### 2.4. Occupational Health Professionals

The undertakings may organize occupational health services, public authorities, official services, social security institutions, and competent authorities based on national conditions and practice. In addition, some countries have regulations relating to occupational health services depending on the size of the enterprise, such as in-plant occupational health services in large-sized enterprises and group services in small-sized enterprises.

1. Occupational health physicians: management activities for employees, including preplacement medical examination, medical surveillance, medical removal, return to work, follow-up, investigation of occupational poisoning or occupational disease, health promotion, post-employment medical examination, and implementation of an occupational health program in the workplace such as periodic education, providing advice on workplace health and safety issues, helping with audit/evaluation of the occupational health program in the workplace, and maintaining the medical records of employees. Table 2 compares the responsibilities of occupational physicians in the US, Malaysia, and Thailand [21,25,26,27].
2. Occupational health nurses: manage cases and provide treatment, follow-up, referrals, and emergency care for occupational injuries and illnesses; counsel workers regarding occupational injuries and illnesses, emotional problems, and substance abuse; promote health and health education; and advise the employer on legal and regulatory compliance, assist in risk management such as collecting health and hazard data, and use the data to prevent injuries and illnesses. According to the Thai Ministry of Health, doctors and nurses must be provided when there are 200 employees or more. For 200 or more employees, one or more nurses (not specific to occupational nurses) are employed during all working hours, and one or more general practitioners (not specific to occupational physicians) are used twice or more per week and 6 h or more per week. For 1000 or more employees, two or more nurses (not specific to occupational nurses) are employed throughout the work period, and one or more general practitioners (not specific to occupational physicians) are used three times or more per week and 12 h or more per week [28]. Table 3 compares the responsibilities of occupational health nurses in the US, Malaysia, and Thailand [21,25,26,27].
3. Safety officers: advising the employer on the safety and health measures in the workplace, inspecting the machinery, plant, and equipment, substance, appliances, and processes used in the workplace that may impact employees’ health, and investigating occupational injuries and illnesses. According to the Thai Ministry of Health, safety officers are regulated, whereas other occupational health professionals are not. The number, types, and responsibilities of safety officers vary depending on the type of enterprise and the number of employees. Furthermore, in addition to the duties of the safety officers mentioned above, the safety officers have responsibilities for hazard identification, risk assessment, advice on safety policies, education, and training of employees for safe work, data collection, and analysis to report occupational health injuries and illnesses [29,30]. Table 4 compares the responsibilities of safety officers in the US, Malaysia, and Thailand [21,25,26,27].

### 2.5. Participatory Action Research

This study aims to establish and apply occupational health services in an enterprise. Because most of the data of this study will be collected in qualitative data to explore the problems by empowering people to share participants’ opinions to find appropriate methods to arrange the difficulties, qualitative research is the most relevant in this study. 

There are many types of qualitative research, but the most appropriate type of qualitative research in this study is participatory action research because participatory action research begins with fundamental problems or issues in societies with limiting self-determination and self-development leading to participatory action. Research develops to create political debate, discussion, and change. Similar to this research, issues at the start need to be solved appropriately. Three types of action research are categorized by different objectives of each type, including:

1. Technical/scientific/collaborative action research;
2. Practical/mutual collaborative/deliberative action research;
3. Emancipating/enhancing/critical science/participatory action research.

Action research of each type has different aspects in the source of concerns, methods, goals, and outcomes. The person who identifies issues varies depending on each type, the researchers in the first type of action research, the researchers and participants in the second type of action research, and the participants with the assistance of researchers in the third type of action research. Each action research’s goals differ from the first type of action research as it tests a particular intervention based on a pre-specified theoretical framework. The second type of action research understands new common problems, causes, and plans for changing processes. The third type of action research explains and resolves actual problems in a specific setting and assists participants in identifying and raising the consciousness of participants’ fundamental problems. The first type of action research outcome is efficient, immediate, but unsustainable change. The second type of action research is immediate enthusiasm and short-lived interventions. The third type of action research is achieved, and sustained changes focus on personal and cultural norms. 

For the reasons mentioned above, the objectives of this study are establishing and applying occupational health services in an enterprise; therefore, participants should participate significantly in this study to identify the problems in the enterprise of the participants until the plan change process is sustainable and the problems are resolved in the awareness of participants. Therefore, this study’s most appropriate action research is participatory action research [31,32,33].

## 3. Materials and Methods

### 3.1. Study Design

**Figure 1:**
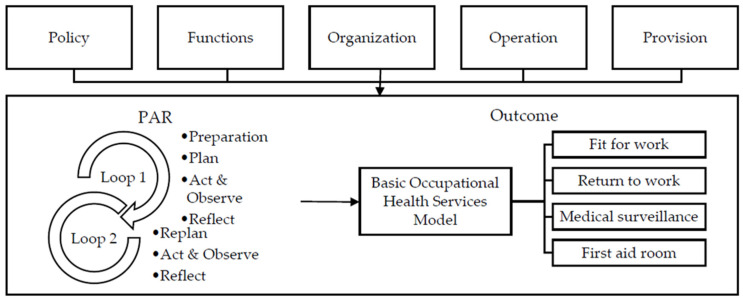
Conceptual framework of PAR cyclical activities.

The selection of an enterprise for the case study was made from large enterprises with more than 200 employees. A large enterprise was selected as a case study for developing occupational health services in the workplace due to (1) the employer’s awareness and willingness to provide financing for OHS. In contrast, the small-to-medium enterprise survey focused more on safety injuries than occupational health. (2) Due to previous experience with occupational health problems, the large enterprise can identify occupational health problems and needs. According to The Ministerial Regulation, Specification of Occupational Safety, Hygiene, and Environment Management Standards (2006), this enterprise size was category 2, including one or more nurses and safety officers. We selected an aluminum production industry in the northeast region [29]. The Conceptual framework of PAR cyclical activities was shown in Figure 1.

### 3.2. Participants and Recruitment

The thirty research participants in this study included: 

The employer, including a manager, participated in analyzing problems and causes and generated action plans based on the focus group discussion to solve problems during the planning phase.

Employees, including 20 sector heads and employees, were the key informants who provided the majority of the information in the focus group discussion on occupational health problems and the occupational health needs of the occupational health service in the analysis of the problem and cause through focus group discussion. They represented those who were assigned to all career fields. The selection of workers, who participated in focus group discussions (FGDs) as key informants, was carried out by snowball sampling. The inclusion criteria of the participants were workers who had a work-related illness, work-related injury, or other health problems or were a client of OHS. The researcher initially contacted the safety officers to inform them of the inclusion criteria for the research participants. As a result, safety officers grouped and appointed sector heads and employees. Next, we recruited participants for the study through interviews in person at the enterprise. Sector heads and workers suggested additional participants based on the inclusion criteria, up to 20.

The occupational safety and health professionals included an occupational physician, three occupational health nurses, and two safety officers. The occupational physician and three occupational health nurses educated and counseled managers, sector heads, and worker representatives in each sector of the occupational health service. Two safety officers provided primary (FGDs) and secondary (documents) data for the problem and cause analysis, and based on focus group discussion, they generated action plans in the planning process. They carried out the assigned action plans as part of the action and observation phase.

The moderator motivated the participants to express their opinions on the problem and caused analysis and development of action plan processes. The inclusion criteria for the moderator were workplace employment, good communication skills, and the ability to communicate with others in the company. In this study, the safety officer was the moderator.

### 3.3. Data Collection

Primary data were drawn from participant observations, walk-through surveys, field notes, focus group discussions, and meeting minutes. Secondary data were extracted from documentation, including industrial hygiene data, safety data sheets, lists of preplacement and periodic medical examinations, reports of occupational injuries and illnesses, OHS provided in the first aid room, illness records, medical unit statistics, the fit-for-work system, return to work assessments, and medical surveillance. In addition, the researchers requested permission to observe and take notes during the focus group discussions. In order to protect the identity and privacy of the participants, no audio, video, or photographs were taken or recorded. Each focus group discussion session took approximately 1 to 1.5 h.

A researcher developed the PAR model in the aluminum production industry in northeastern Thailand between August 2021 and 2022. The PAR cyclical activities included two loops of cyclical movements. The first loop included four phases: (1) the preparation phase; (2) the planning phase; (3) the action and observation phase; and (4) the reflection phase.

### 3.4. Data Analysis 

Data were collected from both primary and secondary data. Primary data were drawn from participant observations, walk-through surveys, field notes, focus group discussions, and meeting minutes. Secondary data were extracted from documentation, including industrial hygiene data, safety data sheets, lists of preplacement and periodic medical examinations, occupational injuries and illnesses reports, OHS provided in the first aid room, illness records, medical unit statistics, the fit-for-work system, return to work assessments, and the medical surveillance system. The data were analyzed using both inductive and deductive thematic analyses. 

#### 3.4.1. Situation Analysis 

This study applied the five key components of occupational health service following the ILO C161 Convention for situation analysis, including (1) policy: objective occupational health service, following national policy, all workers, action plans, and consulted organizations; (2) functions: eleven items of occupational health service functions, (3) organization: provision for establishment, organization, and cooperation; (4) operation: multidisciplinary personnel, inform of health hazards, the working environment, the relationship between ill health and hazards; and (5) provision: authority responsible for supervising and advising occupational health service. Situation analysis compared pre and post participatory action research on occupational health service development [5,10].

#### 3.4.2. Problem and Cause Analysis and Development of Action Plans

This study applied thematic analysis to analyze the problem and cause in phase 2. The thematic analysis processes were (1) starting with data collection, a researcher identified the selected data to analyze; (2) inductive analysis was performed by reviewing, interpreting, and identifying the relationships of the collected data and categorizing common coding into six main themes in loop 1 and 3 main themes in loop 2; and (3) deductive analysis was conducted by comparing the OHS relationships in the ILO C161 Convention.

### 3.5. Participatory Action Research

The first loop included four phases: (1) the preparation phase; (2) the planning phase; (3) the action and observation phase; and (4) the reflection phase. 

#### 3.5.1. Phase 1: Preparation Phase

We reviewed the company’s industrial hygiene data, safety data sheets, preplacement and periodic medical examination lists, occupational injury and illness reports, and OHS provided in the first aid room.

#### 3.5.2. Phase 2: Planning Phase 

A situational analysis was performed using the ILO C161 Convention. A problem and cause analysis was also conducted, including participant observations, a walk-through survey on occupational health risks and problems, and a survey on occupational health illnesses and OHS education provided to workers by OHP and FGDs conducted by moderators. During the research, the participants developed action plans while the researchers observed and took minutes. FGDs were conducted to create action plans and assess the feasibility of those action plans. The researchers then reviewed the action plans of managers, sector heads, workers, safety officers, and human resources workers with an OHP consultant. 

#### 3.5.3. Phase 3: Action and Observation Phase 

A researcher supported the preparation of responsible persons and related documents, such as Thai laws, guidelines, and manuals, for the research participants before implementing action plans. The responsible people carried out the implementation of the action plans. The results and problems that arose during the implementation of the action plans were collected through participant observations and FGDs.

#### 3.5.4. Phase 4: Reflection Phase 

This phase was characterized by summarizing and analyzing the data after implementing the action plans. The resulting information was prepared by one of the researchers. Finally, FGDs were conducted to assess the achievement of the goals and to plan second-loop improvements.

The second loop included three phases: (1) the planning phase using FGDs, after identifying the action plan implementation problems to develop new action plans; (2) the action and observation phase, conducted in the same way as the first loop; and (3) the reflection phase summarizing and analyzing the data after implementing the action plans. Finally, the returned information was prepared by a researcher. Therefore, the development of an OHS model is summarized as follows (Table 5).

## 4. Results and Discussions

### 4.1. Participants’ Demographic Characteristics

The mean age of the participants (n = 30) was 42.18 years. The highest age was for the occupational health nurse job was an age of 58. The participants were classified by job or disease criteria. The job criteria consisted of: (1) the employer; (2) an occupational physician; (3) an occupational health nurse; and 4) a safety officer. The disease criteria comprised: (1) work-related illnesses, including lower back pain and myofascial pain syndrome; (2) work-related injuries, including accidents and foot fractures; and (3) other health problems, including anemia, asthma, clavicle fracture, diabetes mellitus, hypertension, kidney disease, migraine, and thyroid disease. Table 6 describes the demographic characteristics of the participants. 

### 4.2. Situational Analysis

**Figure 2:**
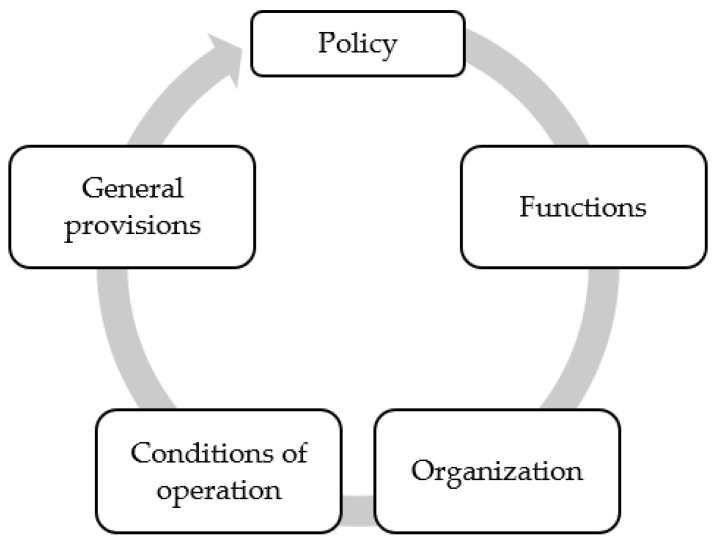
Situational analysis following the ILO C161 Convention.

The main findings of the two loops of PAR are as follows. The research participants included managers, safety officers, human resources workers, heads of sectors, workers, and OHP. During the planning phase, a situational analysis was conducted according to the ILO C161 Convention, including the five components in Figure 2 [5,10].

#### 4.2.1. Policy

The policy of this company is that safety, occupational health, and the environment are the priorities. The policy includes six items, for example that the enterprise will: (1) strictly follow all the relevant laws, regulations, and safety and environment standards; (2) continuously improve the safe work process in order to achieve a safe and healthy working environment; (3) establish a safety, environment, and energy committee (SEE committee); (4) promote, support, train, and assess the risk of safety, occupational health, and the environment to encourage all levels of employees to be aware of working; (5) require all supervisors to supervise work, give advice, coach, and be a role model; and (6) communicate company policies through activities involving employees, stakeholders, and nearby communities. The policy of this company is consistent with all applicable laws and regulations and is comparable to ILO Convention C161. The previous study found the need to maintain and strengthen the worker health and safety policy aiming at the promotion and protection of the worker’s health and consequently, the implementation of positive impact strategies [34].

#### 4.2.2. Functions

Most activities of occupational health, safety, and the environment in 2022 follow policies related to safety activities due to regulations through laws. OHS activities were organized to promote safety activities, including periodic examinations, medical treatment, and prevention. Compared to BOHS, fit for work was changed from not being job specific to a specific job in each department. The list of preplacement examinations was changed from the same medical examination for all workers to a specific department. Return to work was newly developed. Medical surveillance was changed from periodic examination to a medical surveillance program. The first aid room was changed from acute care to first aid, emergency treatment, and emergency preparedness.

#### 4.2.3. Organization

The employers and employees were added to the legal and regulatory provisions to establish OHS. Some hospital-based OHS functions were transferred to an in-plant OHS provider. The employers and employees collaborated in PAR to develop the OHS.

#### 4.2.4. Conditions of Operation

The responsibilities of the safety officers in all activities were changed to the responsibilities of other personnel in the working process. The workers were informed of the health risks and evaluated the relationship between the risks and their work. A new manual was developed in each department to identify the relationship between hazards and health effects. OHP assessed the association between workers, hazards, and health.

#### 4.2.5. General Provisions

Due to the lack of authority at the intermediate level for supervising operations and advising on occupational health services, the company resolved these issues by consulting occupational health professionals who worked at the hospital’s occupational medicine clinic to provide occupational health services, such as health promotion and consultation of abnormal medical examination results. 

### 4.3. PAR Process to Develop Basic Occupational Health Services

#### 4.3.1. PAR Process of Loop 1

The analysis of the problem and cause by the FGDs and participant observation consisted of constructing a research question tool and passing a validity test by three occupational physicians before FGDs. Inductive analysis was applied to the problems and causes by evaluating, interpreting, discovering links, and classifying common coding into six key themes in Table 7.

The process of developing action plans for resolving problems was found through FGDs and the participant observations, based on six key problem themes in Table 8.

Implementation in conjunction with the medical surveillance program includes: (1) a walk-through survey; (2) identifying hazards; (3) industrial hygiene data; (4) significant exposure and health risk assessment; and (5) the design of medical surveillance comprising history, physical examination, and biomarkers of exposure and effect; and (6) medical examination [35,36]. Following the identification of the issues, action plans were developed. The problems, action plans, implementation, and results are shown in Table 9.

#### 4.3.2. PAR Process of Loop 2

The limitations of the sub-branch, the facility’s discomfort, and the document’s inadequacy were recognized through a focus group discussion during the evaluation phase. The development of action plans was carried out in response to the issues listed in Table 10.

### 4.4. Four Basic Occupational Health Service Activities Developments

#### 4.4.1. Fit-for-Work Model Development

OHS activities related to fit-for-work evaluation were developed: indication, medical evaluation, medical certificate, assessor, and medical evaluation result. The indication was an extended job transfer add-on for new workers. The medical evaluation was transformed from the same medical examination despite a different department to the new preplacement evaluation, specifically each department, by an occupational physician, including a preplacement medical evaluation for specific tasks which may involve a danger to worker’s health, e.g., heat and a hot environment [5,10] along with the reasons for performing a fit-for-work evaluation including a preplacement and job transfer [6]. The general medical certificate was changed to a fit-for-work certificate form in each department, including a review of the medical history, a general physical examination, and laboratory tests. The fitness for work was assessed by the occupational physician instead of any doctor. Finally, the result of the medical evaluation was altered from the absence of results after assessment to fit and unfit differently in the fit-for-work opinion, including fit for duty, unfit, and fit subject to accommodation because the company could provide workers with jobs in other departments that were recommended [5]. The development of the fit-for-work model is summarized in Table 11. 

#### 4.4.2. Return to Work Model Development

As a result of legislation, the criteria for when a worker should return to work were changed from “those who had an injury or illness and were on sick leave for more than three days” [37] to “those who had a chronic illness with medical restrictions such as heart disease, lung disease, or brain disease [38], were admitted to the hospital after surgery, frequently took sick days, or were on sick leave for more than three days” along with after a prolonged absence for health reasons, severe illness, or injury according to reasons for performing a fit-for-work evaluation [5,6,10,39]. No change was made to the employee’s return-to-work assessment after the occupational physician detected the indication. The evaluation of returning to work is the responsibility of the occupational physician. A new return-to-work form was created to include additional documentation for medical review. After a return-to-work assessment, the fit-for-work designation was altered from “no management” to temporarily or permanently “fit,” “unfit,” “fit with restrictions,” and “fit with limitations” along with recommending appropriate action to protect the workers and of determining the worker’s suitability for the job and needs for reassignment with the fit-for-work opinion, including fit for duty, unfit, and fit subject to accommodation [5,6,10,34,39]. The fit note should be a recommendation of the fitness for work advice, work modifications, work solutions, working hours and duties adjustment, and equipment, as in previous studies [40,41,42]. The return-to-work location was relocated from a hospital to an enterprise with access to occupational health services. The form for referrals was improved. The development of the return-to-work model is summarized in Table 12.

#### 4.4.3. Medical Surveillance Model Development

After medical evaluations did not improve, a new medical surveillance program was created. Occupational physicians conducted walk-through surveys, identified hazards, and assessed health risks. A manual of hazards and health effects in each department was developed and distributed to workers to monitor early signs of work-related or occupational diseases. No changes were made to the preplacement examination, so a physician developed new baselines for each department. The new medical surveillance program comprised history taking, physical examination, and biological monitoring both of exposure and effect [34]. An occupational physician and nurse will inform workers of their medical exam results with interpretation and management, not in the medical record book. An occupational physician was responsible for confirming diagnoses, determining possible occupational causes, recommending appropriate action, and determining the worker’s suitability for their job [5,10]. The participation of the occupational physician was motivated by the implementation of workers’ health surveillance [43]. Similar to the previous study, medical surveillance contributed to the early identification of diseases related to work or not. It was carried out by an occupational physician to provide examinations for employees at specific times such as periodic examinations and leaves of absence or change of function [34]. The justifications for transformation started with education and learning experience which led to improving the knowledge of workers to identify workers’ occupational health needs. Moreover, feedback on the results was an important process to implement BOHS as in a previous study [44].

The development of the medical surveillance model is summarized in Table 6. Compared with elements of a medical surveillance program, it comprises: 1. a walk-through survey; 2. known hazards; 3. a measurement area or personal sampling; 4. an action level or health risk assessment; 5. the design of medical surveillance programs; 6. medical examinations at regular intervals; 7. the provision of information to employees; 8. the interpretation of the ongoing data analysis of the test; 9. medical removal; 10. a written report; 11. the employee’s work environment re-evaluated as necessary; 12. medical record keeping; 13. audits; and 14. employer actions [35,36]. The new activities were developed according to the components of a medical monitoring program listed in the brackets in Table 13.

#### 4.4.4. First Aid Room Model Development

The steps in the development of the first aid room model were carried out following first aid and the risk management process consisting of: (1) identifying potential causes or needs assessment; (2) assessing the workplace risk; (3) fixing the problems of first aiders and first aid procedures; and (4) reviewing the effectiveness of first aid [45,46]. The 31 first aiders were trained in first aid practices to respond to life-threatening emergencies through a basic life support (BLS) training program (approximately one first aider for every 47 employees) [18,47,48]. The registered nurse employed was responsible for supervising first aid and maintaining the first aid facilities [49]. Applying a triage system in the first aid room procedure and identifying conditions for hospital referral, a new assessment emergency condition was developed from the absence of assessment severity in the first aid room and no identified conditions for hospital referral. This is a recent development, ranging from no assessment after treatment to the nurse being responsible for assessing clinical improvement after a worker’s illness and treatment. The basic life support (BLS) training program was incorporated into developing the new emergency plan. A health promotion program was developed after analyzing the results of a medical exam as part of the problem-solving procedure. The development of the first aid room model is summarized in Table 14.

### 4.5. The in-Plant Basic Occupational Health Service Model

**Figure 3:**
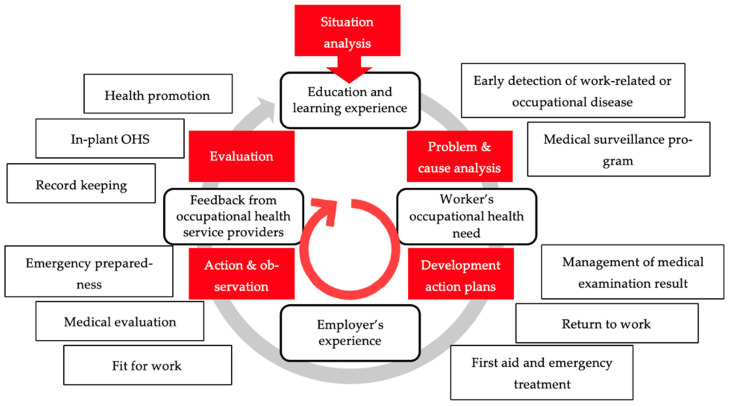
The cycle of participatory action research for developing occupational health services.

The PAR process enabled the participants to share their experiences and collaborate on developing an organizational model for BOHS [13,14]. The important element of PAR to enable factors to develop BOHS was participants perceiving the need to change and be willing to participate in change in the study [50]. The study used education and learning experience to improve occupational health service development [51], as well as workers’ occupational health needs, employers’ experience, and feedback from occupational health service providers, to justify this process. A PAR cycle was developed to describe the process, which begins with a situation analysis and concludes with an evaluation of the replanning to ensure sustainability, as depicted in Figure 3. The education and learning experience enabled workers to help to identify problems in the PAR process [52,53]. The previous studies found that the development of an occupational health culture among workers, creating awareness, establishing existing structures and procedures, and training by both needs assessment and evaluation together is crucial for successful training and long-term sustainable improvements [54,55,56,57]. The PAR process was the tool for key elements of BOHS development that corresponded to key elements of successful health and safety management, including policy, organization, planning, implementation, feedback to enhance BOHS, and auditing [37]. There is an urgent need for community-based strategies that build local agency in the process of describing relevant issues and identifying acceptable solutions, while building towards sustainable policy change over time [58]. The four rectangles in the middle represent the rationale for the transformation, while the rectangle on the outside represents the resulting OHS activities. After education and experience, a system for early detection and medical surveillance was developed. OHS activities, including management of medical examination results, return to work evaluation, and first aid and emergency treatment, were developed in response to occupational health needs. The employer’s experience led to OHS activities that included fit-for-work evaluation, medical evaluation, and emergency preparedness. In response to the feedback from the OHS provider, OHS activities were developed that included health promotion, OHS in the plant, and recordkeeping. Following the steps in the BOHS activity process, education and learning experience in the PAR process along with information and education, medical surveillance consisted of both work environment surveillance and worker’s health surveillance, early detection of work-related or occupational diseases along with the diagnosis of occupational and work-related diseases, emergency preparedness, and first-aid treatment, and the development of medical records [17].

### 4.6. Multidisciplinary Staff

Multidisciplinary teams should have clearly defined roles and collaborate on various tasks to provide BOHS in the workplace. Employee education and prevention are the responsibility of occupational health and safety professionals, such as industrial hygienists, industrial engineers, and safety professionals. Safety professionals are responsible for developing procedures, standards, and systems to control and reduce hazards and exposure. Only health professionals, on the other hand, can treat illness and injury beyond first aid. Occupational physicians and occupational health nurses certified in occupational medicine have the skills and competencies for educational training in epidemiology, toxicology, industrial hygiene, recognition and management of occupational illnesses and injuries, research, and general management of a comprehensive occupational health program [25,41,59,60].

Occupational health professionals are responsible for occupational health activities. The responsibility for safety activities is shared with the safety officer. Laws govern occupational health providers, such as physicians, nurses, and safety officers. According to law, the occupational physician is responsible for the preplacement examination, the periodic examination, and return-to-work [21,41].

#### 4.6.1. Occupational Physicians

Currently, occupational physicians are not regulated like consultants at work or for workplace examinations because laws states that an agreement can be made with a nearby hospital for treatment instead of workplace examinations [19]. According to the findings of this study, unless an examination is conducted following the law, the core competency of an occupational physician in BOHS is specifically preventive functions such as fit-for-work assessment, return-to-work assessment, medical surveillance by designing a medical surveillance program, and a first aid room for medical emergency preparedness and health promotion [21,25,26,27].

#### 4.6.2. Occupational Health Nurses

Nurses, not occupational health nurses, regulate the number of nurses on the job, but no one is responsible for the OHS function. Nurses in the workplace are responsible for first aid and emergency treatment. However, they do not have a preventive function due to a lack of interaction with managers and the safety committee or authority to effectively recommend appropriate preventive measures such as worker counseling and health education programs [21,25,27,28].

#### 4.6.3. Safety Officers

Both the number of safety officers and their duties in the workplace are regulated by law. The duties of safety officers have been defined in terms of safety and occupational health [30]. According to the study of 26 necessary competencies and the proficiency of safety officers in Thailand, the employer expected safety officers to perform safety and occupational health activities [61]. According to the current study’s findings, occupational health activities should require recommendations from occupational health professionals and safety officers to achieve practical implementation of occupational health activities [25,27].

## 5. Conclusions and Recommendations

### 5.1. Conclusions

**Figure 4:**
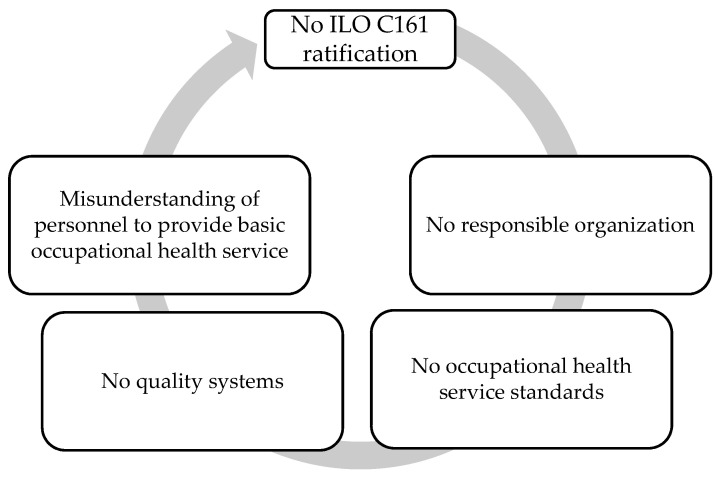
The cycle of limitations to developing occupational health services.

PAR improves the basic occupational health service model by bringing stakeholders together to identify needs and experiences, develop action plans, and implement solutions. The study’s findings include a better understanding of the problem and its causes in Thai enterprises and suggestions for future development in similar settings. Figure 4 shows the limitations of creating OHS. There is (a) no ratification of ILO C161, (b) no responsible organization to provide educational and training programs for the qualification, certification, and competencies of occupational health professionals, (c) no law-regulated occupational health service standards, (d) no responsible organization has implemented quality evaluation methods for OHS in enterprises, and (e) a misunderstanding that the provision of basic occupational health services is the responsibility of safety officers.

**Figure 5:**
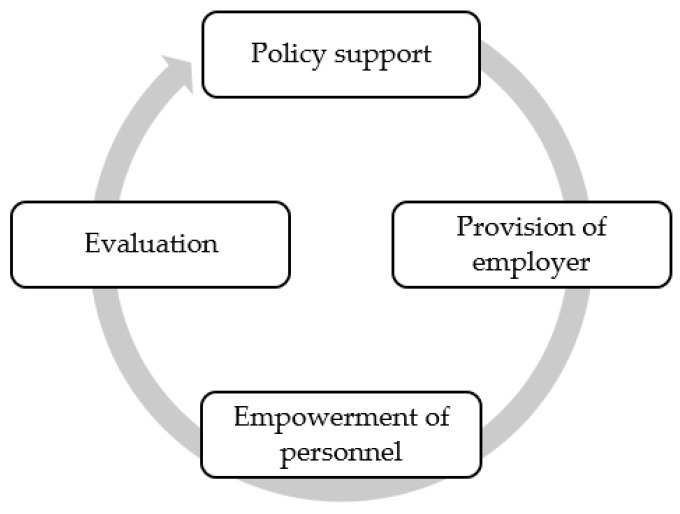
Organizational factors to sustainably develop occupational health services.

Figure 5 shows the organizational factors required for the sustainable development of OHS: (a) policy support: OHS activities were carried out according to OHS policy, (b) the employer’s provision: the employer was aware of fundamental occupational health issues and addressed their root causes, (c) education and learning experiences were crucial tools to empower personnel in OHS development to identify occupational health problems and occupational health needs [17], and (d) OHS development planning and continuity evaluation.

### 5.2. Recommendations

The recommendations will be implemented with limitations to develop OHS at national and enterprise levels. Limitations to creating a national OHS include (a) no ILO C161 ratification, (b) no responsible organization to provide educational and training programs for occupational health professionals’ qualification, certification, and competencies, (c) no occupational health service standards regulated by law, (d) no responsible organization has implemented quality evaluation methods for OHS in enterprises, and (e) a misunderstanding that providing basic occupational health services is the responsibility of safety officers. However, the future of OHS laws remains unpredictable, and enterprise- concerned policy may be an additional OHS provision at work. BOHS can be developed by the organizational factors required for the sustainable development of BOHS comprised of (a) policy support: OHS activities are conducted according to OHS policy, (b) employer provision: the employer is consciously aware of fundamental occupational health issues and resolves their root causes, (c) education and learning experiences are crucial tools to empower personnel in the development of OHS for identifying occupational health problems and occupational health needs [17], and (d) OHS development planning and continuity evaluation.

Recommendations for the enterprise include: (a) developing BOHS following the ILO C161 Convention under the policy, (b) contacting the hospital’s occupational medicine clinic to provide and counsel for the development of occupational health services, (c) conducting internal audits to ensure continuous development of OHS and, (d) identifying OHP as duties in the working process. According to the stepwise development of occupational health services, there is step II: BOHS infrastructure that varies according to local conditions and needs for developing BOHS content. The occupational physician and occupational health nurse provide BOHS with the support of a safety officer with knowledge and experience in accident prevention and basic safety [17].

National recommendations include: (a) ratifying the ILO; (b) establishing responsible organizations for training, qualification, and certification; (c) implementing national OHS laws and standards following the ILO C161 Convention, which clarified occupational health service functions and occupational health professional duties; and (d) organizations for auditors, such as the Healthcare Accreditation Institute (a public organization) for auditing hospital settings, which should be strengthened and standardized. According to the stepwise development of occupational health services, step III: international standard service is the minimum objective for each nation as mandated by the ILO C161 Convention. The content of OHS is predominantly preventive, although curative services can also be provided appropriately. Multidisciplinary personnel, especially occupational physicians, should have specialized training from specialized units (such as an institute of occupational health) [17].

## Figures and Tables

**Table 1:** The infrastructure system of occupational health services.

Level	Responsible Organizations	Responsible Duties
National level	MOH/MOL DOH&S DOH	Regulation and policy
Intermediate level	Institute of occupational health, Occupational medicine clinic (university/hospital)	Support service, e.g., training, consultation, certification, research, and development
Local level	Tertiary referral center In-plant/in-house OHS SOCSO occupational health clinic Public occupational health clinic OHS group practice center Private occupational health clinic Private clinics	Providing occupational health service

Note: The infrastructure system of occupational health services at the national level. Adapted from “Basic occupational health services—their structure, content, and objectives”.

**Table 2:** Duties of occupational physicians.

US	Malaysia	Vietnam
- Clinical, occupational, and environmental medicine - Law and regulations - Environmental health - Work fitness and disability management - Toxicology - Hazard recognition, evaluation, and control - Disaster preparedness and emergency management - Health and human performance - Public health, surveillance, and disease prevention - Management and administration	- Preplacement examination and medical surveillance - Analysis of occupational diseases and poisoning co-related with risk assessment - Interpretation and explanation of the results of investigations and recommendation of further action - Notification of occupational diseases and poisoning to DOSH and the employer - Conducting occupational health programs - Providing education and advice on health and safety issues - Audit/evaluation of occupational health programs - Maintaining medical records	- Performing preplacement examinations such as fitness for work within 30 days - Performing periodic examinations such as medical surveillance at least yearly - Performing physical examinations during other periods such as the early detection of occupational disease - Performing return-to-work medical assessments when an employee stops working for three days or more

**Table 3:** Duties of occupational health nurses.

US	Malaysia	Thailand
- Identifying potential hazards and finding ways to prevent, eliminate, minimize, or reduce hazards - Training programs to promote workplace health and safety - Enhancing the accuracy of OSHA record keeping. - Providing screening related to specific chemicals or exposure, including preplacement physical examinations, job placement assessments, periodic inspections, and maintenance of confidential employee health records - Managing work-related illnesses and injuries, emphasizing early recognition and intervention; making recommendations about work restrictions or removal; and following up and monitoring workers as they return - Health promotion programs - Guiding case management of employees with prolonged or complex illnesses and injuries	- Managing cases—treatment, follow-up, referrals, and emergency care for job-related injuries and illnesses - Counseling and intervening in crisis; counseling workers about related illnesses and injuries, substance abuse, and emotional problems - Promoting education programs that encourage workers to take responsibility for their health - Advising employers on legal and regulatory compliance - Assisting in risk management, e.g., gathering and using health and hazard data to prevent injury and illness	- No identification in laws, regulations, or standards

**Table 4:** Duties of safety officers.

US	Malaysia	Thailand
- Focusing on developing procedures, standards, or systems to control or reduce hazards and exposure detrimental to people, property, and/or the environment	- Advising employers on measures in the interests of the safety and health of persons employed - Inspecting the machinery, plant, equipment, substances, appliances, or process or any description of manual labor used in place of work, that is of such nature liable to cause bodily injury to any person working - Investigating any accident, near-miss accident, dangerous occurrence, occupational poisoning, or occupational diseases	- Following laws, regulations, and guidance - Analyzing possible dangers - Assessing safety risks - Analyzing work plans and projects on safety measures - Inspecting and assessing workplace operations - Training on work safely - Examining and appraising working conditions - Management of occupational safety - Analyzing and investigating causes of accidents, illness, or annoyance at work - Compiling statistical data and analyzing and writing reports on accidents, illness, or annoyance - Education about occupational and environmental disease for employees - Conducting other occupational and safety activities

**Table 5:** Summary of this study’s loops, stages, activities, and session plans.

Loops	Phases	Description	Sessions
Loop 1	Preparation	- List of large enterprises that can participate. - Inviting a large enterprise to participate. - Contacting an occupational health professional to educate the participants. - Collaboration team: core researcher and moderator. - Data collection from the documentation: industrial hygiene, safety data sheets, lists of preplacement and periodic medical examination, occupational injuries and illness reports, and OHS provided in the first aid room	3
	Planning	- Situational analysis per ILO C161 - Informed consent processing of participants and agreement on the time and place for regular sessions - Education for workers on the topic of occupational health illnesses and occupational health services by occupational health professionals - Participant observation using a walk-through survey - FGDs with workers conducted by moderators for the problem and cause analysis of occupational health problems and occupational health services - Identifying and categorizing occupational health problems by using thematic analysis of six key themes comprising: 1. the association between hazards and health effects; 2. indications of OHS; 3. lists of medical examination; 4. management of medical examination results; 5. attitudes to occupational health professionals; and 6. facilities and documents - FGDs to develop action plans including: 1. defining the association between the work and illnesses; 2. identifying the indications of OHS; 3. the completeness of lists of medical examination; 4. clarifying working process to provide occupational health; 5. identifying the duties of occupational health providers; and 6. identification of essential facilities and documents	6
	Action and observation	- The researcher prepared the participants and the related documents, including Thai laws and regulations, national standards, and Thai standards - OHP walk-through survey - The OHP identifies health hazards, significant exposure assessment, health risk assessment, list of preplacement, and periodic medical examination in the walk-through survey report - The OHP provides a manual of hazards and health effects in each department - FGDs to assess an appropriate list of pre-placement and periodic medical examination and to improve the completeness of the manual of the hazards and health effects in each department - FGDs on the objectives of what indications are in each BOHS and the identification duties of occupational health providers in flowcharts and tables in each BOHS - FGDs on the objectives of the working process in each BOHS and essential facilities and related documents	3
	Reflection	- The limitations of the sub-branch, the discomfort of the facility, and the inadequacy of the document were recognized through FGDs - The development of new action plans was conducted in response to the issues	3
Loop 2	Re-planning	- The action plans comprised: (1) approval lists of medical examination by the main branch; (2) return-to-work evaluation in the enterprise; and (3) completeness of referral forms	2
	Action and observation	- The enterprise provided a comparative price per person between the current and new medical examinations. The enterprise provided information on the reasons for changing to a new medical examination. The enterprise offered new medical examinations to the main branch enterprise - Safety officers offered occupational physicians the opportunity to assess RTW in the enterprise. The occupational physicians accepted the opportunity to carry out an assessment of RTW in the enterprise. Safety officers grouped the indicated workers and appointed occupational physicians to assess RTW in the enterprise - The safety officers consulted with an occupational physician to suggest additional patient data before going to an occupational physician to improve the referral form. The occupational physician suggested additional patient data. The safety officers improved the referral forms following suggestions by the occupational physicians	3
	Reflection	- FGDs to establish the participatory action research cycle for developing occupational health services - Occupational safety and health professionals recommended for further OHS development	3

**Table 6:** Participants’ demographic characteristics.

Job/Disease Criteria	Job Title	Disease	ID	Age (Year)	Year of Service (Year)	Sex
Job	Manager	-	1	45	20	Male
	Occupational physician	-	2	41	7	Female
	Occupational health nurse	-	3	58	35	Female
	Occupational health nurse	-	4	56	33	Female
	Occupational health nurse	-	5	43	20	Female
	Safety officer	-	6	41	18	Male
	Safety officer	-	7	35	12	Female
	Safety officer	-	8	25	2	Female
Disease	Employee	Accident	9	33	10	Male
	Employee	Accident	10	49	28	Female
	Employee	Anemia	11	46	11	Male
	Employee	Anemia	12	49	13	Male
	Employee	Asthma	13	39	12	Female
	Employee	Clavicle fracture	14	38	18	Male
	Employee	Diabetes mellitus	15	45	13	Female
	Employee	Diabetes mellitus	16	42	14	Male
	Employee	Foot fracture	17	32	6	Male
	Employee	Hypertension	18	39	8	Female
	Employee	Hypertension	19	44	13	Female
	Employee	Hypertension	20	48	28	Female
	Employee	Hypertension	21	45	28	Female
	Employee	Hypertension	23	41	13	Male
	Employee	Kidney disease	25	46	13	Female
	Employee	Low back pain	26	34	12	Female
	Employee	Low back pain	27	48	27	Female
	Employee	Migraine	28	41	13	Male
	Employee	Myofascial pain syndrome	29	35	7	Male
	Employee	Thyroid disease	30	43	18	Male

**Table 7:** The process of problems and causes analysis.

Problems	Participants	FGDs
1. Association between hazards and health effects	Manager	- What diseases or illnesses may be aggravated or worsened after exposure to hazards in each department?
	Employees	- My illness was caused by work or it was not.
	Employees	- I do not know how hazards in my work affect my health or aggravate my illness.
2. Indications of OHS	Safety officer	- I am uncertain what medical conditions should be assessed during return-to-work.
3. Lists of medical examination	Sector heads	- We had the same medical examination despite being in different departments.
	Employees	- Preplacement medical examinations and periodic medical examinations were not appropriate for their jobs.
	Employees	- When they obtained work adaptations, they only received periodic examinations in their current job. As a result, they suspect that the hazards in their old department may affect their health.
4. Management of medical examination result	Employees	- I did not know the meaning of my medical examination results.
	Employees	- I did not know how to manage abnormal medical examination results. As a result, I was not treated when I abnormal medical examination results.
5. Attitude to occupational health professionals	Sector heads	- I returned to work despite my clinical condition not improving after I received medical treatment in the first aid room.
	Employees	- I was assessed for my return to work after I became sick. I desired reassurance from a doctor or nurse that my health meant that I was fit for work and that the hazards and risks in my workplace would not affect my health.
	Employees	- I returned to work, although my illness was not resolved, and it deteriorated after returning to work.
6. Facilities and documents	Employees	- Inadequate medicine and medical equipment in the first aid room.
	Employees	- No medical record from the workplace when I went to hospital.

**Table 8:** The process of developing action plans.

Problems	Participants	FGDs
1. Defining the association between work and illnesses	Safety officer	- I suggested that the occupational physician should prepare the manual of medical examination in each department, including hazards with health effects, to reassure the workers.
2. Identifying the indications of OHS	Safety officer	- I suggested that fit-for-work indications should be applied to both new employees and employees who obtain work adaptations.
	Safety officer	- I offered to establish the criteria for return to work.
3. Completeness of lists of medical examination	Manager	- I suggested that occupational physicians should identify appropriate preplacement lists and periodic medical examinations in each department.
4. Clarifying the working process to provide occupational health	Occupational physician	- The triage system should be applied in the first aid room to categorize the severity of the medical condition and to refer the worker to hospital. If necessary, the nurse will refer the worker to hospital with a medical record form.
5. Identifying duties of occupational health providers	Manager	- I suggested that the occupational physician should identify the list of medical examinations in each department.
	Safety officer	- I suggested the duties of the occupational physician, including the assessment of fit-for-work status before the worker starts working and the assessment of the worker before they return to work.
	Safety officer	- Occupational physicians are responsible for diagnosing and treating workers with abnormal medical examination results and providing medical examination and treatment in the first aid room to assess the lack of medicine and medical equipment.
	Safety officer	- Nurses are responsible for assessing the association between worker illnesses and their job—consulting occupational doctors for occupational disease diagnosis. In addition, when the workers have illnesses, nurses are responsible for assessing their clinical improvement after treatment.
6. Identification of essential facilities and documents	Safety officer	- Establish a medical record form for the workplace.

**Table 9:** Problems, action plans, implementation, and results of loop 1.

Problems	Action Plans	Implementation	Results
1. Association between hazards and health effects	Defining the association between work and illnesses	1. OHP walk-through survey. 2. OHP identifies health hazards, significant exposure assessment, health risk assessment, and lists of preplacement and periodic medical examinations in the walk-through survey report. 3. OHP provides a manual of each department’s hazards and health effects. 4. FGDs to improve and assess the completeness of the manual of hazards and health effects in each department.	Components of the manual of the hazards and health effects in each department: 1. Hazards and health effects 2. Health risks assessment 3. Health conditions may be worsened or aggravated 4. Lists of preplacement medical examination 5. Lists of periodic medical examination 6. FFW certificate in each department
2. Indications of OHS	Identifying the indications of OHS	1. Preparation of related documents before FGDs. 2. FGDs on the objectives of what indications there are in each BOHS.	1. Indications of fit-for-work 2. Indications of return-to-work 3. Indications of medical surveillance 4. Indications of first aid room
3. Lists of medical examination	Completeness of lists of medical examination	1. OHP walk-through survey. 2. OHP identifies health hazards, health risk assessments, and lists of preplacement and periodic medical examinations. 3. The researcher prepared the objectives and the interpretation in each list of medical examinations. 4. FGDs to assess the appropriate list of preplacement and periodic medical examinations.	1. Lists of preplacement medical examination 2. Lists of periodic medical examination
4. Management of medical examination results	Clarifying the working process to provide occupational health	1. FGDs on the objectives of the working process in each BOHS.	Components of the flowchart: 1. Methods 2. Description 3. Related documents 4. Responsible person 5. Duration
5. Attitude to occupational health professionals	Identifying duties of occupational health providers	1. Identification duties of occupational health providers in the flowchart and table in each BOHS.	Duties of occupational health professionals
6. Facilities and documents	Identification of essential facilities and documents	1. The enterprise provided data on the medical unit providing services. 2. Preparation of related documents before FGDs. 3. FGDs on the objectives of essential facilities and documents.	1. Lists of medication 2. Lists of medical equipment 3. Lists of documents

**Table 10:** Problems, action plans, implementation, and results of loop 2.

Problems	Action Plans	Implementation	Results
Limitation of the sub-branch	Approval lists of medical examinations by the main branch	1. The sub-branch enterprise provided a comparison price per person between current and new medical examinations. 2. The sub-branch enterprise provided information on reasons for changing to the new medical examination. 3. The sub-branch enterprise offered the main branch enterprise new medical examinations.	The main branch enterprise approved lists of medical examinations
Inconvenience of the facility	Return to work in the enterprise	1. The safety officers offered the occupational physicians the opportunity to assess RTW in the enterprise. 2. The occupational physician accepted the offer to complete the assessment of RTW in the enterprise. 3. Safety officers grouped the indicated workers and appointed occupational physicians to assess RTW in the enterprise.	The occupational physician assessed RTW in the enterprise
Incompleteness of documents	Completeness of referral forms	1. Safety officers consulted occupational physicians to suggest additional patient data before going to the occupational physician for an improvement referral form. 2. The occupational physicians suggested additional patient data. 3. The safety officers improved the referral form following the suggestions of the occupational physician.	Development of referral forms

**Table 11:** Fit-for-work model development.

Component	Old Activities	PAR	Justifications	New Activities
Indication	New workers	Problem analysis	Worker’s occupational health need The workers did not undergo medical examinations after work adaptations	1. New worker 2. Job transfer
Medical evaluation	Same medical examination despite the different department	Problem analysis	Employer’s experience The managers want to know contraindicated medical conditions in each department, particularly for highly sensitive jobs	1. New preplacement evaluation, specifically each department, by the occupational physician 2. Preplacement medical evaluation for heat and hot environments form
Medical certificate	General medical certificate	Development of action plans	Support procedure Providing additional documents for the medical evaluation	Fit-for-work certificate form in each department
Occupational physician	Not-fit-for-work assessment by the occupational physician before working	Problem analysis	Employer’s experience In high-risk jobs, the managers experienced workers being ill while working	The occupational physician assesses the fitness to work
Result	No results after medical assessment	Problem analysis	Employer’s experience In high-risk jobs, the managers experienced workers being ill while working	The results of the medical evaluation were divided into fit and unfit

**Table 12:** Return-to-work model development.

Component	Old Activities	PAR	Justifications	New Activities
Indication	Employees on sick leave ≥ 3 days	Problem analysis	Education and learning experience Awareness of conditions should be assessed by RTW	1. Chronic disease with medical restrictions such as heart disease, lung disease, and brain disease 2. Hospital admission or after surgery 3. Frequent sick leave 4. Sick leave ≥ 3 days
Occupational physician	No return-to-work assessment	Problem analysis	Worker’s occupational health needs No RTW assessment after illness. The workers desired reassurance from doctors or nurses that their health, fit-for-work status, and their work would not affect their health	The occupational physician assesses the return-to-work employee who has identified an indication
Return to work form	No return-to-work form	Development of action plans	Worker’s occupational health needs Providing additional documents convenient for medical evaluation	Return-to-work form
Result	No management after the return-to-work assessment	Problem analysis	Worker’s occupational health needs No RTW assessment after illness. The workers desired reassurance from doctors or nurses that their health, fit-for-work status, and their work would not affect their health	Result: fit, unfit, fit with restriction, and fit with limitation
Place	The occupational physician assessed the return-to-work status in the hospital	Problem analysis II	Feedback from occupational health service providers The safety officers offered the occupational physicians the opportunity to assess RTW in the enterprise	The occupational physician assessed the return-to-work status in the enterprise
Referral form	Incompleteness of the referral form	Problem analysis II	Feedback from occupational health service providers The occupational physicians explained that the RTW problem was due to the incompleteness of the referral form.	The safety officers improved the referral form following the suggestions of the occupational physician

**Table 13:** Medical surveillance model development.

Component	Old Activities	PAR	Justifications	New Activities
Design a medical surveillance program	No improvement in periodic medical evaluation	Problem analysis	Worker’s occupational health needs The periodic medical evaluation was not appropriate for their jobs	Occupational physician 1. Walk-through survey (1) 2. Identify hazard (2) 3. Bring measurements (3) 4. Significant exposure and health risk assessment (4) 5. Design a medical surveillance program (5)
Medical surveillance program (6)	Medical surveillance program	Problem analysis	Education and learning experience Workers suspected their illnesses were caused by work or not and how hazards in their work affect their health and aggravate their illnesses	1. New medical surveillance program: - History taking - Physical examination - BEI of exposure - BEI of effect 2. Manual of the hazards and health effects in each department: - Hazards and health effects- Health conditions may be worsened or aggravated
Medical examinations	No preplacement and more frequent examination	Problem analysis	Employer’s experience The managers would like to know the contraindications of medical conditions in each department, specifically highly sensitive jobs	- New preplacement evaluation including baseline specifically for each department by the occupational physician
Provide test results to employees (7)	Provide in information in medical record books	Problem analysis	Worker’s occupational health needs 1. The workers suspected an association between their abnormal medical examination results and their jobs 2. The workers did not know the meaning of my medical examination results	Occupational physicians and nurses inform workers of their medical examination results with interpretation and management
Management of the results of medical surveillance (9)	Re-evaluate the work environment following the medical examination results	Problem analysis	Worker’s occupational health needs The workers did not know how to manage their medical examination results	1. The occupational physician is responsible for reconfirming, diagnosing, and treating workers with abnormal results. 2. Safety officers re-evaluate the work environment (11)

**Table 14:** First aid room model development.

Component	Old Activities	PAR	Justifications	New Activities
Assessment of emergency condition	-No assessment severity in the first aid room -No identified conditions for referral to hospital	Problem analysis	Worker’s occupational health needs What conditions should you go to hospital?	-Assess the emergency condition -Triage system in the first aid room -Conditions for referral to hospital
Assessment of clinical improvement	No clinical assessment after treatment	Problem analysis	Worker’s occupational health needs Workers came back to work despite no clinical improvement after receiving medical treatment	After the workers suffered illnesses, the nurse was responsible for assessing their clinical improvement after treatment
Emergency plans	No emergency plans	Development of action plans	Employer’s experience Managers need workers who can provide life support in emergencies	Basic life support training program
Health promotion	No health promotion program	Problem analysis	Worker’s occupational health needs Management of abnormal medical examination results	Health promotion program for hypertension

## Data Availability

Data are available upon request.
